# Lectin-Based Fluorescent Comparison of Glycan Profile—FDA Validation to Expedite Approval of Biosimilars

**DOI:** 10.3390/ijms25179240

**Published:** 2024-08-26

**Authors:** Sarfaraz K. Niazi, Sesselja Omarsdottir

**Affiliations:** 1 College of Pharmacy, University of Illinois, Chicago, IL 60612, USA; 2 Faculty of Pharmaceutical Sciences, University of Iceland, IS-107 Reykjavik, Iceland; sesselo@hi.is

**Keywords:** antibodies, biosimilars, FDA, lectins, microarray, fluorescent monitoring

## Abstract

Glycan profile comparisons are one of the most tedious analytical exercises for establishing compliance with recombinant therapeutic protein batches. Based on its intensive research, the FDA has confirmed that lectin array binding with fluorescent monitoring is the fastest and most reliable method for profile comparisons. Using a database of over 150 biological products expressed in nine diverse mammalian cell systems, the FDA immobilized 74 lectins to study their binding using fluorescently labeled glycoproteins. The FDA identified nine distinct lectins from a custom-designed lectin microarray: rPhoSL, rOTH3, RCA120, rMan2, MAL_I, rPSL1a, PHAE, rMOA, and PHALs, which detect core fucose, terminal GlcNAc, terminal β-galactose, high mannose, α-2,3-linked sialic acids, α-2,6-linked sialic acids, bisecting GlcNAc, terminal α-galactose, and triantennary structures, respectively. This method can be used for screening and routine testing and to monitor batch-to-batch variability of therapeutic proteins, including establishing analytical similarity as a crucial part of biosimilar development.

## 1. Introduction

**Figure 1:**
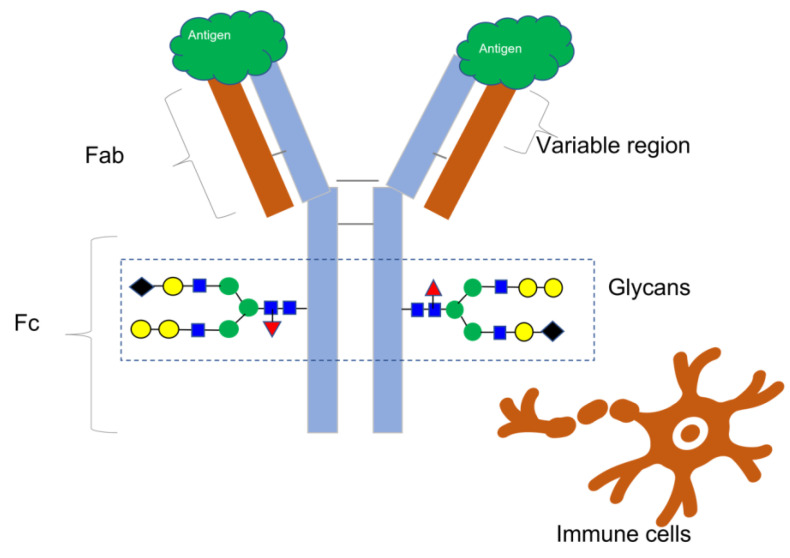
This illustration depicts the basic structure of an antibody, featuring two indistinguishable heavy chains (colored blue) and two indistinguishable light chains (colored brown) that combine to form a Y-shaped configuration. The varied domains of the Fab region account for the antibody’s exceptionally precise affinity for an epitope on the surface of an antigen. The Fc region of the antibody engages with different immune cells to facilitate immunological reactions. The addition of various carbohydrate molecules (shown by colored geometric shapes) to each heavy chain forms glycans, which have a significant impact on Fc-mediated processes such as antibody-dependent cell-mediated cytotoxicity and other immune effector mechanisms. Therefore, glycans are essential quality features to consider when evaluating antibody-based treatments.

Since the approval of muromonab-CD3 (brand name Orthoclone OKT3) in 1986 [1], monoclonal antibodies (mAbs) and antibody-based treatments have become integral to modern medicine, accounting for nearly 160 approved novel molecular entities [2]. These consist of bioengineered antibody types, antibody–drug conjugates, and monoclonal antibodies. While nearly half of these drugs are used to treat cancer, they are also essential in the treatment of other illnesses, such as viral infections like HIV and Ebola and immune-mediated disorders [3]. Most therapeutic mAbs belong to the IgG class, comprising four protein subunits that form a “Y”-shaped molecule with matching pairs of heavy and light chains, as Figure 1 illustrates. The unique amino acid arrangement in the variable regions of the heavy and light chains at the ends of the Y arms determines an antibody’s binding specificity to a target molecule, also known as an antigen. An antigen has a distinct structural domain, called an epitope, to which an antibody binds [4].

The National Cancer Institute defines an antigen as any substance that elicits an immune response, including toxins, chemicals, bacteria, viruses, or other exogenous substances. Additionally, body tissues and cells, including cancer cells, bear antigens capable of triggering immune responses [5] (Figure 1).

Therapeutic proteins exhibit various types of glycosylation, primarily N-linked and O-linked, each of which impacts protein function differently. N-linked glycosylation occurs at asparagine residues within the consensus sequence N-X-S/T (where X can be any amino acid except proline), involving complex oligosaccharide chains attached to the protein backbone via an N-glycosidic bond. This glycosylation type is essential for protein folding and stability, influencing the protein’s biological activity and half-life in circulation. In contrast, O-linked glycosylation occurs on serine or threonine residues, contributing to mucin-like properties that affect protein solubility and mucosal interactions [6,7].

Antibodies undergo significant alterations in glycosylation, beginning at a specific amino acid on the Fc section of each heavy chain, where carbohydrate molecules are attached one after another to create branched structures called glycans. Glycans in antibody treatments can impact antibody–receptor interactions on different immune cells, influencing functions such as antibody-dependent cell cytotoxicity and patient immune responses [6]. Newly developed glycans in bioengineered antibodies can potentially elicit negative immune reactions, making N-glycans crucial in determining therapeutic protein quality and affecting their stability, safety, and effectiveness [6]. Variations in the glycosylation profiles of mAbs can result from genetic sequences utilized for protein expression in cells, the specific circumstances maintained during cell culture (such as nutrition levels, pH, oxygen availability, and cell density), and the purification techniques employed. Hence, consistently monitoring glycosylation patterns throughout production from upstream to downstream stages, during scale-up, and across several batches is essential to maintaining uniform product quality.

Antibody production typically involves mammalian cell culture systems, requiring careful control over glycosylation and other post-translational modifications to ensure consistency and efficacy. Regulatory considerations reflect these differences; analytical similarity assessments of biosimilars are rigorous, including characterization of glycosylation patterns, structural integrity, and functional similarity compared to a reference product. Similar principles apply to non-antibody biologics, but specific analytical and clinical comparability requirements may differ based on the type of biologic and its intended therapeutic use [8].

Factors influencing glycosylation in therapeutic proteins are multifaceted and critical to the efficacy and safety of these biologics. The choice of expression system plays a significant role, as mammalian cells typically produce proteins with complex glycosylation patterns resembling human glycoproteins. In contrast, microbial systems like bacteria and yeast may result in different glycosylation profiles. Proteins derived from natural sources, such as human plasma, inherently possess native glycosylation patterns. Additionally, glycosylation can be engineered or modified in therapeutic proteins to enhance pharmacokinetics, stability, or other properties. For biotechnologically derived proteins, analysis of glycosylation patterns is crucial, with standard techniques based on high-performance liquid chromatography, mass spectrometry, and capillary electrophoresis [9].

Glycans or carbohydrates attached to therapeutic glycoproteins must be adequately analyzed and controlled throughout the product life cycle due to their impact on product quality, safety, and efficacy. The complexity of protein glycosylation poses a significant analytical challenge, involving attaching complex oligosaccharide chains to specific amino acid residues, which impacts protein folding, stability, and interactions with other molecules in the biological system [10]. Glycan structures attached via N-linked or O-linked glycosylation pathways vary widely across therapeutic proteins, contributing to their functional diversity and therapeutic efficacy. Understanding glycosylation is pivotal in biologics development, particularly in biosimilars, where demonstrating similarity in glycan profiles to the reference product is crucial for regulatory approval [8]. The effect of glycosylation on antibody functionality and pharmacokinetics is well understood, and glycoengineering strategies that enhance the pharmacokinetic properties of therapeutic proteins offer new approaches to optimize therapeutic efficacy [11].

Monoclonal antibodies (mAbs) exemplify therapeutic proteins in which glycosylation critically influences biological activity and therapeutic efficacy [12]. For instance, the glycans attached to the Fc region of mAbs regulate interactions with Fc receptors and complement proteins, affecting effector functions such as antibody-dependent cellular cytotoxicity (ADCC) and complement-dependent cytotoxicity [13].

Non-antibody biologics, like enzymes, growth factors, cytokines, and hormones, can be produced using bacterial, yeast, or mammalian cells. These biologics have various structures and functions and work through enzymatic activity or receptor binding mechanisms. While glycosylation is essential for antibody function, affecting their stability, pharmacokinetics, and immunogenicity, non-antibody biologics show different glycosylation patterns depending on the expression system used. This variation impacts their stability, solubility, and receptor-binding affinity [10,14].

Understanding the glycosylation of antibody and non-antibody therapeutics is essential for ensuring efficacy, safety, and regulatory compliance. The acceptance of robust and fast analytical methods for glycan analysis is crucial to expedite the approval of biological drugs, particularly biosimilars, for process control and to determine batch-to-batch variability in biological manufacturing.

## 2. Comparative Glycosylation

The complexity of current analytical techniques employed for describing glycan structures presents challenges, often involving extracting the N-glycans from the protein structure using different separation processes and mass spectrometry to determine the exact molecular weights of the carbohydrate molecules [15,16]. However, technological advances have improved glycosylation characterization in biologics, ensuring more accurate analytical similarity assessments of biosimilars [17]. Advanced analytical methods for glycosylation profiling, such as mass spectrometry, (U)HPLC, and capillary electrophoresis, have become more sophisticated, enabling detailed characterization of glycoproteins. These methods provide comprehensive glycan structural and compositional information but are complex and time consuming [18].

Comparative glycosylation studies are crucial for biosimilarity assessments [19], emphasizing the significance of understanding glycosylation-dependent immunogenicity and its implications for safety and efficacy [20]. N-glycosylation is vital for the stability, safety, immunogenicity, efficiency, and serum half-life of glycoprotein biologics. It is a critical quality attribute that must be rigorously monitored during development, manufacturing, and batch release.

Advanced analytical techniques, including (U)HPLC, mass spectrometry, and capillary electrophoresis, are widely used to characterize glycosylation patterns. (U)HPLC separates and quantifies glycan structures based on their size and composition, providing detailed information on glycan heterogeneity. MS offers high sensitivity and specificity in identifying glycan structures, enabling precise glycosylation profile characterization by comparing glycan masses and fragmentation patterns [21]. Using peptide-N-glycosidase (PNGase F) to treat the glycans and remove the N-glycans from the protein backbone is one of the most commonly used methods for glycan testing. This is followed by fluorophore dye labeling and LC or LC-MS detection [22]. Another common technique is CE, which separates glycans according to their size and charge, providing a high-resolution method for profiling intricate glycosylation patterns [23]. Other less common techniques for glycan characterization and routine biologic testing include nuclear magnetic resonance (NMR) spectroscopy, high-performance anion-exchange chromatography with pulsed amperometry detection (HPAEC-PAD), and capillary electrophoresis with laser-induced fluorescence detection (LIF) [24,25].

Together, these techniques form a comprehensive toolbox for characterizing and analyzing glycosylation in biosimilar development, ensuring robust comparability with the reference product [24].

## 3. Lectin-Based Testing

Lectin-based assays complement these methods, offering valuable insights into glycosylation differences or similarities between glycoprotein samples. They are especially valuable for monitoring the manufacturing process, comparing batch-to-batch differences, and assessing the analytical similarity of biosimilars [22,23,26,27,28,29,30]. Lectins, a heterogeneous group of naturally occurring or genetically engineered proteins, share a crucial feature with antibodies: the ability to bind to specific glycan structures or epitopes, like how antibodies attach to antigens [31].

These assays quantitatively analyze specific glycan structures by measuring lectin binding signals. They help determine whether a biosimilar maintains acceptable glycosylation patterns compared to reference products, contributing to the robust scientific and regulatory evaluation of the analytical similarity of biosimilars. For accuracy, results from lectin-based assays are correlated and validated with additional techniques like (U)HPLC-MS or CE to confirm glycan identities and quantities [32]. Establishing acceptance criteria for analytical similarity assessment involves predefined glycosylation parameters that align with regulatory guidelines and the scientific literature.

Lectin-based assays complement chromatographic and MS methods, providing qualitative insights by selectively binding to specific glycan epitopes on therapeutic proteins and providing quantitative and qualitative insights into glycosylation motifs relevant to protein function and immunogenicity [23]. They play a pivotal role in the biosimilarity assessments of therapeutic antibodies by enabling a targeted analysis of specific glycosylation patterns, allowing comparisons between reference and biosimilar antibodies. Similarities or differences in glycosylation profiles can be identified using a panel of lectins with known specificities for different glycan structures, such as high mannose and sialic acid; similarities or differences in glycosylation profiles can be identified [33].

An integrated approach combines lectin-based assays with other analytical and functional methods to comprehensively demonstrate the analytical similarity of biosimilars. These assays must meet regulatory requirements from agencies like the U.S. Food and Drug Administration (FDA) or European Medicines Agency (EMA) regarding the comparability and similarity of glycosylation profiles [34].

The benefits of lectin-based assays in biosimilarity assessments include their specificity for glycan structures, allowing for targeted analysis of glycosylation patterns relevant to antibody function and efficacy. Their sensitivity enables the detection of subtle differences in glycosylation profiles that may impact antibody behavior. By comparing lectin binding profiles between reference products and biosimilar antibodies, researchers can establish confidence in biosimilarity concerning glycosylation [28,33].

Additionally, chemiluminescent lectin-binding assays facilitate faster detection of differences in antibodies, ensuring the consistency and quality of therapeutic proteins, especially in the context of biosimilars [35]. This integrated and rigorous analytical framework, incorporating lectin-based assays, is essential for robust scientific and regulatory evaluation of biosimilarity.

## 4. The FDA Initiative on Lectin-Based Microarrays for Glycan Profiling

To meet the requirement for an efficient and fast technique to assess glycosylation patterns in antibody-based therapies and to overcome the limitations of previous lectin-based assays, particularly their lack of selectivity for specific glycans, which resulted in false-positive or inconclusive signals [23], researchers at The Center for Drug Evaluation and Research (CDER) have recently investigated the potential of lectin microarrays using so-called LecChip-IgG-mAb for the detection of glycan epitopes in therapeutic antibodies [9].

**Figure 2:**
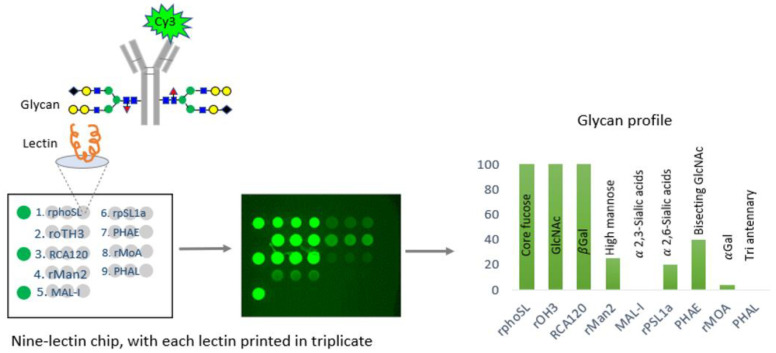
An illustration of the technique devised by CDER researchers for efficiently analyzing glycosylation patterns in manufacturing monoclonal antibodies (mAbs). This method utilizes lectins, a group of proteins that selectively bind to different glycan structures in mAbs. CDER’s research has discovered nine lectins, each with a specific affinity for one of nine commonly occurring epitopes in mAb therapies. Subsequently, these lectins were fixed onto a glass chip. The tested glycosylated antibodies are marked with a fluorescent molecule called Cy3. By incubating them on the nine-lectin chip and using a fluorometer to measure the fluorescence intensity of each lectin-containing well, one can obtain quantitative data regarding the unique glycan epitopes on the antibody [26,36].

In a comprehensive study, the CDER examined data from over 150 FDA-approved therapeutic antibody product applications submitted to the FDA’s Electronic Common Technical Document (eCTD) system between December 1994 and May 2023. These antibodies were produced using various mammalian cell expression systems, leading to the identification of nine universal glycan epitopes common across all therapeutic monoclonal antibodies. To study these epitopes, the researchers immobilized 74 unique lectins on glass chips, each with a specific affinity for different glycan epitopes. By incubating these lectins with fluorescently labeled monoclonal antibodies (mAbs) known for their glycan structures, the researchers identified nine distinct lectins that selectively bind to the nine common epitopes. An integrated approach combines lectin-based assays with other analytical and functional methods to demonstrate the analytical similarity of biosimilars [29]. A comprehensive microarray consisting of nine lectins was created and used to confirm the specificity of each lectin for a certain glycan. This was accomplished by conducting binding experiments using commercially available innovative and biosimilar antibodies with well-documented glycosylation profiles and two therapeutic proteins that do not have glycosylation. In addition, the precise binding of each of the nine lectins was verified by employing glycosidases, which are enzymes that eliminate carbohydrate groups from the glycans. The binding specificity of each lectin was further confirmed using glycosides, enzymes that remove carbohydrate groups from glycans, verifying the alterations in binding that corresponded with the removal of specific epitopes. This microarray successfully identified distinct glycosylation patterns aligned with proprietary data for the reference product antibody (infliximab) and its three biosimilars [26] (Figure 2).

The CDER-developed lectin microarray offers a significant advantage over conventional analytical glycan methods by allowing the determination of intact glycoprotein samples, thus eliminating inaccuracies associated with sample handling, and the separation of glycans from the protein structure of glycan epitopes in all FDA-approved monoclonal antibody (mAb) products [26]. Therefore, the CDER approach for evaluating the glycosylation of antibody-based therapies has the potential to be widely applicable to novel therapeutic products. This FDA study presented a specially developed lectin microarray that included nine different lectins: rPhoSL, rOTH3, RCA120, rMan2, MAL_I, rPSL1a, PHAE, rMOA, and PHAL, which detect core fucose, terminal GlcNAc, terminal β-galactose, high mannose, α-2,3-linked sialic acids, α-2,6-linked sialic acids, bisecting GlcNAc, terminal α-galactose, and triantennary structures, respectively [26]. The lectins were designed to bind only to the typical N-glycan epitopes in therapeutic IgG antibodies. The microarray consisting of nine lectins allows for the efficient and fast analysis of glycans, which has the potential to be used for high-throughput screening, enabling comparisons of glycosylation patterns using intact glycoprotein samples [26].

Furthermore, the lectin microarray approach has been successfully applied in the CDER investigation on different types of glycosylated and non-glycosylated therapeutic proteins (cytokines, IgG1, IgG1-fusion protein, IgG2, and IgG4), as well as to compare the glycan profiles of infliximab and three of its biosimilars [26]. These studies demonstrated the microarray’s capability to detect subtle glycosylation differences, such as variations in high mannose and sialylated glycan levels, thereby supporting biosimilarity claims [26].

The lectin-based assays primarily generate semi-quantitative data, providing insight into the relative abundance of specific glycan structures. While the lectin microarray effectively profiles glycan patterns, its results are typically correlated or validated using more quantitative techniques, such as MS and CE, for initial glycan characterization [26].

Despite the advantages of the lectin microarray, the method has some limitations. The semi-quantitative nature of the data and potential variability in lectin-chip quality may require additional validation. Moreover, the method may need to fully distinguish between closely related glycan structures, which could necessitate further analysis using conventional analytical techniques. However, its ability to analyze intact proteins and detect subtle glycosylation changes makes it a valuable tool in the screening and routine monitoring of glycosylation and for analytical similarity comparisons between biosimilars and reference products [26].

The protocol utilized in the CDER paper is described in [23]. The robustness of this particular lectin microarray must be demonstrated across various matrices, including different salt concentrations, variable pH buffers, and cell culture media, making it versatile for biopharmaceutical contexts. The method’s accuracy, precision, robustness, repeatability, linearity, and specificity vary depending on the specific lectins used and the assay conditions and are a part of the assay validation for each product. 

However, a study by Fotinopoulou et al. (2003) investigated two lectin-binding approaches to monitor the glycosylation of human monoclonal antibodies during their development and manufacture. The carbohydrate composition was evaluated across various preparations derived from different host cell types, cell sublines, or batches made of the same cells. Lectin binding was quantified using ELISA and surface plasmon resonance (SPR). For comparison, the monosaccharide content of several preparations was also measured using high-pressure anion exchange chromatography (HPAEC) with pulsed amperometric detection (PAD). Both lectin-based methods detected changes in glycosylation when the antibodies were prepared using different methods. SPR showed greater sensitivity than ELISA for some lectins, while ELISA was more sensitive for others. Generally, the lectin results were consistent with those obtained from monosaccharide analysis. However, lectin-based methods were particularly effective in detecting changes in N-acetylneuraminic acid, which HPAEC/PAD could not assess due to the low level of this compound. The lectin-based approaches also offered advantages, such as faster performance, less need for specialized expertise, and the ability to quickly identify glycan structures that might be missed by monosaccharide analysis. It is suggested that initial monosaccharide and/or oligosaccharide profiling should be performed during the development of therapeutic proteins. However, a lectin-based approach is recommended for the routine screening of production batches. Among the two lectin methods, SPR is much faster for screening purposes, while ELISA is beneficial for comparing specific carbohydrate features across multiple samples of the same glycoprotein [27].

Furthermore, in a study by the FDA, the glycan profiles of proteins were investigated using surface plasmon resonance (SPR) to analyze their interactions with lectins. The FDA validated this method by systematically removing sugar moieties from human transferrin using specific glycosidases, demonstrating that protein glycans could be identified based on their selective affinity for different lectins. This technique was further applied to analyze the Fc glycosylation patterns of therapeutic peptibodies and antibodies produced in mammalian cells, specifically CHO and HEK 293 6E cells, with *E. coli*-produced proteins as controls. The study found that antibodies expressed in HEK 293 6E cells contained sialic acid, whereas those expressed in CHO cells did not. Additionally, antibodies from CHO cells exhibited higher fucosylation levels than those from HEK 293 6E cells [30]. The FDA also used this SPR-based approach to quantify the fucose content in glycan-engineered mouse antibodies and determine the mannose content in human antibody variants with altered high-mannose levels. The glycan profiles obtained through SPR were consistent with those derived from the normal-phase separation of 2-AB-labeled glycans. In addition, the binding activity of glycan-engineered antibodies to Fc gamma receptors corresponded with their glycan profiles. These results indicate that SPR lectin binding analysis can be a rapid and effective alternative for characterizing protein glycosylation cells [30].

The N-linked glycosylation of four batches of a commercially available therapeutic mAb was assessed using three orthogonal chromatographic methods and a lectin microarray. N-glycans were enzymatically removed with PNGase F and analyzed through high-pH anion-exchange chromatography with pulsed amperometric detection (HPAEC-PAD) using 21 N-glycan standards. A multi-stage linear gradient allowed for the sequential analysis of neutral and sialylated glycans in a single run. Further analysis involved hydrophilic interaction liquid chromatography with fluorescence detection (HILIC-FD) after labeling the glycans with the fluorescent tags 2-AA and 2-AB, producing distinct separation profiles for orthogonal identification. In parallel, glycan profiling with the lectin microarray required partial denaturation of the mAbs to expose the N-glycans in the Fc region. Fluorescently labeled antibodies against human IgG Fc were used to generate glycosylation fingerprints, which were analyzed to obtain semi-quantitative data on glycan structural classes. Across all four analytical methods, the predominant N-linked glycans were core-fucosylated asialo diantennary structures with varying galactosylation levels. Minor amounts of afucosylated and bisected glycans were also detected, but no sialylation was observed. The mAb demonstrated a high level of consistency in glycosylation profiles across all batches, with a variation coefficient of less than 6%, underscoring the reliability of these techniques in glycosylation analysis [22].

In this lectin microarray study, antibody samples were incubated with lectins at either room temperature or 4 °C, followed by thorough washing to remove unbound lectins. Detection methods varied based on the type of lectin used—enzyme-linked lectins were detected via colorimetric or chemiluminescent methods, while fluorescently labeled lectins were visualized using microscopy or plate readers. The generated signals were analyzed to quantify the presence and relative abundance of specific glycan structures. Controls were included to ensure binding specificity and minimize background noise. These combined approaches provided complementary and corroborative insights into the glycosylation patterns of the mAb, demonstrating the utility of integrating lectin microarray with conventional chromatographic methods for comprehensive glycan analysis [30].

In summary, the lectin microarray technique provides a simple and efficient method for rapidly analyzing glycan profiles. It enables comparisons of glycosylation patterns in intact glycoprotein samples and can process numerous samples quickly. This capability is particularly valuable for assessing glycosylation across different manufacturing batches of biologics and for comparing biosimilars with reference products during development and manufacturing.

## 5. Conclusions

Glycosylation is an essential characteristic of therapeutic antibodies and specific non-antibody therapeutic proteins. It requires comprehensive investigation and careful management at all stages of development and throughout the product’s lifespan. The lectin microarray, developed by researchers at CDER, offers a simple, fast solution and has an excellent capacity for processing large amounts of data. Consequently, it can improve the development and production processes of these increasingly essential treatments [23].

Traditional methods of characterizing glycan patterns or profiles of antibodies typically involve more comprehensive and sophisticated analytical techniques. In practice, both lectin-based assays and traditional analytical methods complement glycan analysis. Lectin-based assays are often used for initial screening or routine monitoring due to their simplicity and speed. At the same time, traditional analytical methods are employed for in-depth structural characterization and validation in complex biosimilar development processes.

Lectin-based assays selectively target specific glycan structures using lectins with known binding specificities. They offer a relatively simple and straightforward method that provides qualitative and semi-quantitative data on specific glycan types and can be performed in a high-throughput manner [33].

However, these assays are limited to the glycan structures recognized by the available lectins and are semi-quantitative at best, with exact glycan composition and linkage details not fully resolved [37]. By contrast, traditional methods such as mass spectrometry (MS), liquid chromatography (LC), capillary electrophoresis (CE), and nuclear magnetic resonance (NMR) provide detailed information on glycan composition, linkage, and modifications [38]. These techniques offer a comprehensive structural analysis of glycans, enabling precise quantification of individual glycan species and identifying rare or unexpected glycan variants. However, they require specialized equipment and expertise, are more time consuming and expensive, and require larger sample quantities, more involved sample preparation processes, and higher purity. When comparing the two approaches, lectin-based assays offer targeted insights into specific glycan motifs but are limited by lectin specificity. Traditional methods provide a broader and more detailed structural analysis, allowing for precise quantification and resolution of glycan structures and offering insights into heterogeneity and modifications that lectin-based assays may miss. In establishing biosimilarity, lectin-based assays provide a quick initial assessment, but traditional methods are crucial for the detailed characterization required to demonstrate comparability in glycosylation profiles and for meeting regulatory expectations [39].

The FDA has shown that the lectin microarray is useful in evaluating glycosylation in different manufacturing batches or comparing biosimilar and reference goods. This demonstrates its potential to speed up the development of biosimilars. The FDA´s conclusions significantly impact the understanding of characterization versus comparison. The key issue is maintaining the glycan profile, as found in clinical lots, as a measure of biosimilarity.

A chemiluminescence-based lectin-binding assay can quickly identify the glycosylation status of antibodies. This test is suitable for the high-throughput evaluation of therapeutic glycoproteins [29].

By integrating these approaches, the industry can ensure that therapeutic antibodies will maintain consistent and effective glycosylation profiles, enhancing their safety, efficacy, and regulatory compliance.
